# Diversity of alkane hydroxylase genes on the rhizoplane of grasses planted in petroleum-contaminated soils

**DOI:** 10.1186/s40064-015-1312-0

**Published:** 2015-09-18

**Authors:** Shun Tsuboi, Shigeki Yamamura, Toshiaki Nakajima-Kambe, Kazuhiro Iwasaki

**Affiliations:** National Institute for Environmental Studies (NIES), Center for Regional Environmental Research, 16-2 Onogawa, Tsukuba, 305-8506 Japan; National Institute for Environmental Studies (NIES), Center for Environmental Biology and Ecosystem Studies, 16-2 Onogawa, Tsukuba, 305-8506 Japan; Faculty of Life and Environmental Sciences (Bioindustrial Sciences), University of Tsukuba, 1-1-1 Tennodai, Tsukuba, 305-8572 Japan

**Keywords:** Bacterial alkane hydroxylase genes, Grass roots, Petroleum contamination, Phytoremediation, Culture-independent molecular approaches

## Abstract

The study investigated the diversity and genotypic features of alkane hydroxylase genes on rhizoplanes of grasses planted in artificial petroleum-contaminated soils to acquire new insights into the bacterial communities responsible for petroleum degradation in phytoremediation. Four types of grass (*Cynodon dactylon*, two phenotypes of *Zoysia japonica*, and *Z. matrella*) were used. The concentrations of total petroleum hydrocarbon effectively decreased in the grass-planted systems compared with the unplanted system. Among the representative alkane hydroxylase genes *alkB*, *CYP153*, *almA* and *ladA*, the first two were detected in this study, and the genotypes of both genes were apparently different among the systems studied. Their diversity was also higher on the rhizoplanes of the grasses than in unplanted oil-contaminated soils. *Actinobacteria*-related genes in particular were among the most diverse alkane hydroxylase genes on the rhizoplane in this study, indicating that they are one of the main contributors to degrading alkanes in oil-contaminated soils during phytoremediation. *Actinobacteria*-related *alkB* genes and *CYP153* genes close to the genera *Parvibaculum* and *Aeromicrobium* were found in significant numbers on the rhizoplanes of grasses. These results suggest that the increase in diversity and genotype differences of the *alkB* and *CYP153* genes are important factors affecting petroleum hydrocarbon-degrading ability during phytoremediation.

**Electronic supplementary material:**

The online version of this article (doi:10.1186/s40064-015-1312-0) contains supplementary material, which is available to authorized users.

## Background

The exploration, extraction, refining, transport, and use of petroleum and derivative products has resulted in soil pollution with petroleum hydrocarbons, which is of critical environmental concern worldwide (Khan et al. 2013). Techniques for cleaning these soils include physicochemical/chemical treatments such as chemical oxidation using ferrous compounds and soil thermal desorption (Langbehn and Steinhart 1995; Ferguson et al. 2004), but these are expensive and environmentally invasive (Pandey et al. 2009; Segura et al. 2009). Biological remediation methods using plants (that is, “phytoremediation”, a green technology) have been recognized as excellent alternatives (Khan et al. 2004; Jain et al. 2011).

Grasses and legumes have been selected and used for phytoremediation of petroleum-polluted soils because of their tolerance to petroleum pollution. Grasses in particular are regarded as candidate plants for efficient phytoremediation because they have fibrous roots (Kaimi et al. 2007) that can loosen soil aggregates and effectively introduce oxygen, which is needed to activate alkanes by terminal oxidation by alkane hydroxylases (van Beilen et al. 2003), along root channels from the atmosphere (Adam and Duncan 1999; Merkl et al. 2005).

A primary concept of phytoremediation is that the petroleum-degrading microorganisms in the rhizosphere, which consists of rhizoplanes (the external surface of roots) and soil close to roots, have their degradation activity enhanced by exudates from the plant roots (Kuiper et al. 2001) and by molecular oxygen introduced from the atmosphere (Adam and Duncan 1999; Merkl et al. 2005). Although previous studies reported that these plants effectively reduced petroleum concentration in the contaminated soils, presumably via stimulation of petroleum-degrading bacteria, the bacterial communities involved in the remediation remain largely unknown. Thus, characterization of the petroleum hydrocarbon-degrading bacteria on the rhizoplanes is indispensable to understanding the phytoremediation mechanisms and improving the efficiency of remediation. This study aims to acquire novel insights into the community structures and diversity of alkane-degrading bacteria on the rhizoplanes of grasses, based on culture-independent molecular approaches.

## Methods

### Plant species

Four types of grass were used in this study: two Japanese lawngrasses [*Zoysia japonica* Steud. and drought-resistant *Z. japonica* Steud. (described as “dr-*Z. japonica*” in this paper)], Manilagrass (*Z. matrella* Merr.), and Tifton Bermuda grass (*Cynodon dactylon* Pers.) were used in this study. The carpeting grasses were obtained from commercial gardening stores.

### Soil preparation, plant experiment and sampling

To compare the diversity and phylogeny of alkane-degrading bacteria among the rhizoplane samples of the four grasses planted under the same experimental conditions, petroleum-contaminated soils (10,000 mg/kg) were prepared by mixed commercial river sands and oil obtained from an actual petroleum-polluted site in Yamaguchi, Japan, in experimental containers (height, 500 mm; width, 600 mm; depth, 800 mm; and volume, 240 L). To increase the water- and nutrient-holding capacity of the soils, they were covered by a 50-mm layer of commercial Akadama soil (small: 2–6 mm diameter, Makino, Tochigi, Japan). Sections measuring 100 mm × 100 mm (length × width) were periodically cut from the 400 mm × 600 mm carpeting grasses for sampling, and the roots sampled were stored at −20 °C for molecular analysis after removing the petroleum-contaminated sands. The contaminated soils were collected to measure total petroleum hydrocarbon (TPH) concentration. Total petroleum hydrocarbon from the polluted soils was extracted and measured as soon as possible (see below). Samples collected at 856 or 891 and 494 days into the study were used to analyze *alkB* genes and *CYP153* genes, respectively.

### DNA extraction from roots and detection of four alkane degradation genes (*alkB*, *almA*, *CYP153* and *ladA*)

DNA of root-associated bacteria was extracted from about 0.2 g of each root sample of the carpeting grass using the FastPrep^®^ instrument and the FastDNA^®^ spin kit for soil (Qbiogene, Carlsbad, CA, USA) according to the manufacturer’s protocol. The PCR reaction was performed with the PCR reaction mixture containing PCR buffer with MgCl_2_, 0.25 mM deoxynucleotide triphosphate, 0.05 U Ex Taq^®^ polymerase (Takara Bio, Shiga, Japan), 2 μM of each specific primer (Table 1), 2 μL of template DNA and nuclease-free water to a final volume of 10 μL, using the Takara Thermal Cycler Dice^®^ Gradient and Takara Thermal Cycler Dice^®^ Touch (Takara Bio). The respective thermal conditions are shown in Additional file 1: Table S1. Successful amplification of the target genes was confirmed by electrophoresis through a 2.0 % agarose gel and 0.5 mg/L ethidium bromide before a cloning procedure.

**Table 1:** PCR primers used in this study

Target gene	Primer name	Sequence (5–3′)	References
*alkB*	AlkB3F	TAYGGNCAYTTCTWYRTYGAGCA	Paisse et al. (2011)
	AlkB3R	GRATTCGCRTGRTGRTC	
*almA*	AlmAdf	GGNGGNACNTGGGAYCTNTT	Wang and Shao (2012)
	AlmAdr	ATRTCNGCYTTNAGNGTCC	
*CYP153*	CYP153-F1	ATGTTYATYGCNATGGAYCCN	Wang et al. (2011)
	CYP153-R2	GCGRTTVCCCATRCARCGRTG	
*ladA*	ladAFR	GGCGTSTACGMCRWCTACGGYRGG	Lo Piccolo et al. (2011)
	ladARV	GAYCTACCAGGYCGGGTCGTCG	
Vector	M13 primer M4	GTTTTCCCAGTCACGAC	Takara Bio
	M13 primer RV	CAGGAAACAGCTATGAC	

### Clone library constructions, sequencing and phylogenetic analysis of *alkB* and *CYP153* genes

Successfully amplified target genes were cloned into the pMD20-T vector with Mighty TA-cloning Kit (Takara Bio) according to the manufacturer’s protocol. The constructed vectors were transformed into *Escherichia coli* JM109 competent cells (Takara Bio). The selected colonies were checked by direct PCR using the vector primers M13 primer M4, and M13 primer RV (Table 1) and Quick Taq™ HS DyeMix (Toyobo, Osaka, Japan) if they had an insert fragment of the correct size. From each sample, about 50 *E. coli* JM109 colonies with the PCR fragment of the correct size were selected randomly and used in further sequencing analysis. The positive fragments were sequenced using the BigDye^®^ Terminator kit v.3.1 (Applied Biosystems, Carlsbad, CA, USA) and the vector primers as above, and the sequences were obtained on an Applied Biosystems 3730 DNA Analyzer (Applied Biosystems). We used BLASTx to perform a homology search of the cloned *alkB* and *CYP153* gene sequences against the GenPept database at the National Center for Biotechnology Information (NCBI). Distance matrices were calculated based on DNADIST of PHYLIP (PHYLogeny Inference Package) 3.695 (http://evolution.genetics.washington.edu/phylip.html) and were used to group the obtained sequences into operational taxonomic units (OTUs) with a distance cut-off of 0.15 (*alkB*) and 0.07 (*CYP153*) using Mothur (Schloss et al. 2009). Rarefaction curves were calculated using “R” statistics software (R Development Core Team, version 2.15.2). Evolutionary distance dendrograms were constructed by the maximum likelihood method with the Molecular Evolutionary Genetics Analysis (MEGA) 6 software package (Tamura et al. 2013). Confidence of the dendrogram topology was evaluated using bootstrap analysis with 100 resamplings.

### Real-time quantitative PCR (qPCR) assay

The standard samples of the target gene quantification were constructed from retrieved and cloned DNAs from the petroleum-contaminated soil and its PCR products. The qPCR was carried out using a Takara Thermal Cycler Dice^®^ Real Time System Single (Takara Bio) and KOD SYBR^®^ qPCR Mix (Toyobo) according to the manufacturers’ protocols. The thermal conditions for *alkB* qPCR were as follows: initial denaturation at 98 °C for 2 min, followed by 40 cycles of 98 °C for 10 s, 55 °C for 15 s and 68 °C for 30 s. For *CYP153*, the same thermal conditions were used with the difference that the final phase at 68 °C was extended to 1 min. All analyses were carried out in triplicate on each extracted DNA sample.

### Analytical method

Total petroleum hydrocarbon was extracted from 0.2 g of sampled soil (wet weight) with 10 mL of polychlorotrifluoroethylene (H-997; Horiba, Kyoto, Japan) as the extraction solvent. Total petroleum hydrocarbon in the solvent was quantified with an oil-measurement instrument OCMA-350 (Horiba, Japan) based on infrared absorption analysis. A standard mixture of OCB (Horiba, Japan) consisting of 2,2,4-trimethylpentane, hexadecane and benzene was used as standard. Total petroleum hydrocarbon concentration per dry weight soil (g) was calculated and converted to mg/kg units from the actual TPH concentration data and moisture ratios of soil samples.

### Nucleotide sequence accession numbers

The nucleotide sequences of the partial *alkB* and *CYP153* genes obtained in this study have been deposited into the DDBJ/EMBL/GenBank databases under the following accession numbers: LC019409 through LC019680 for the *alkB* genes, and LC019154 through LC019408 for the *CYP153* genes.

## Results

### Decrease in total petroleum hydrocarbon (TPH) concentration

**Fig. 1:**
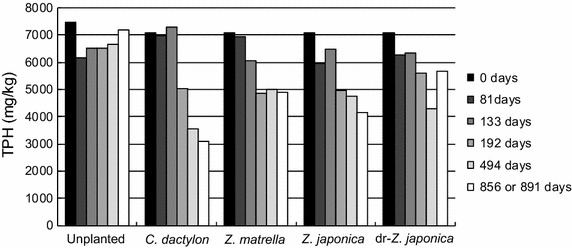
Time course of total petroleum hydrocarbon (TPH) concentration in planted and unplanted systems

Total petroleum hydrocarbon concentration in the soils showed decrease in all planted systems during 856 or 891 days, respectively, while it was nearly unchanged in the unplanted system (Fig. 1). The degree of TPH concentration decrease was different among the planted systems. The *C. dactylon*-planted system in particular decreased in TPH concentration from approximately 7000 mg/kg to approximately 3000 mg/kg.

### Genotypic diversity of cloned *alkB* and *CYP153* genes

**Fig. 2:**
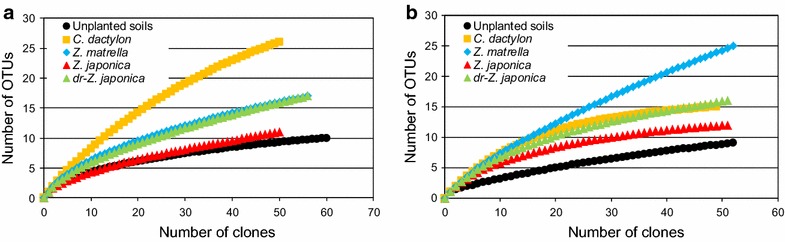
Rarefaction curves of retrieved **a** *alkB* genes at 856 days (*C. dactylon* and *Z. japonica*) or 891 days (unplanted soils, *Z. matrella* and dr-*Z. japonica*) and **b** *CYP153* genes at 494 days from four rhizoplane samples and unplanted contaminated soil samples. The* y axis* shows the number of OTUs grouped at 85 % (*alkB*) and 93 % (*CYP153*) similarity

Of the four alkane hydroxylase genes, *alkB*, *CYP153*, *almA* and *ladA*, the first two were detected from the rhizoplane samples. Cloned 272 *alkB* gene sequences were grouped into 54 OTUs. Rarefaction analysis was carried out based on these OTUs (Fig. 2a). The diversity of cloned *alkB* sequences was higher in the rhizoplane samples than in the unplanted oil-contaminated soil samples. Cloned 255 *CYP153* gene sequences were grouped into 44 OTUs, although four of the sequences obtained were positioned outside the outgroup (*Pseudomonas putida linC*, accession No. AAA25810). As well as *alkB* genes, rarefaction analyses indicated that the genotypic diversity of cloned *CYP153* genes was also higher in the rhizoplane samples than in the unplanted oil-contaminated soil samples (Fig. 2b). These observations were also supported by the diversity parameters (Additional file 1: Table S2).

### Phylogenetic analyses of the *alkB* and *CYP153* genes

**Fig. 3:**
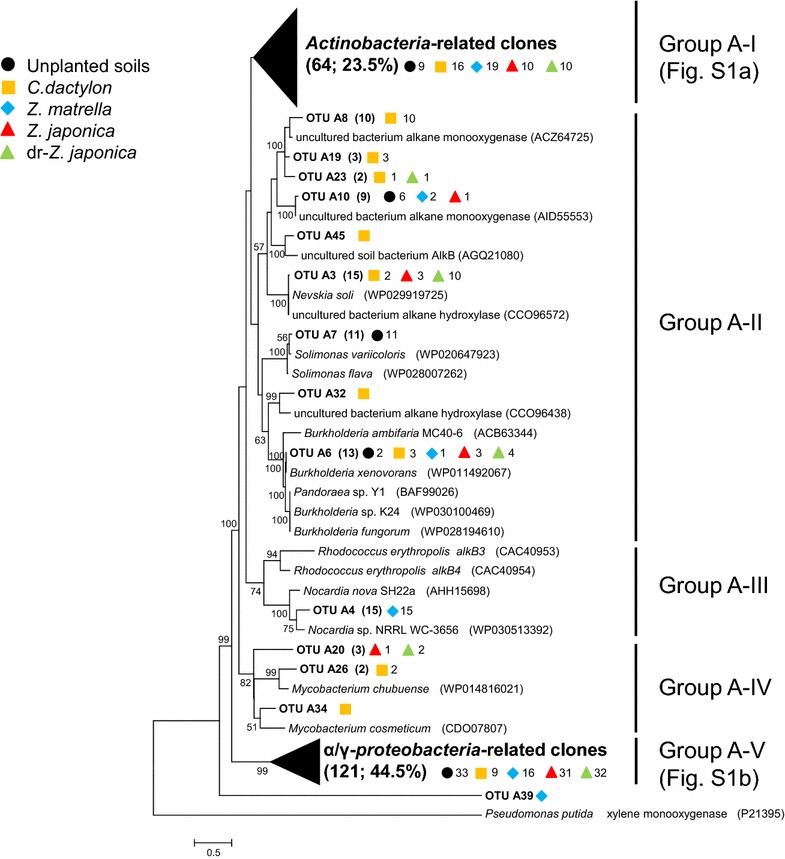
Evolutionary distance dendrogram of retrieved *alkB* sequences with the reference sequences from NCBI database based on OTU grouping. *Numbers* in *parenthesis* show the numbers of sequences affiliated to the same OTU and group. *Symbols* are used to distinguish different clone libraries. *Numbers* on the *right-hand* of the *symbols* reflect the numbers of sequence within each clone library. Bootstrap values below 50 % are not shown

*alkB* OTUs were phylogenetically divided into five groups (Fig. 3). Group A-I, which accounted for 23.5 % of total *alkB* clones, was composed of *Actinobacteria*-related clones. Additional file 1: Figure S1a shows phylogenetic distribution in group A-I in more detail, indicating that this group consisted of various Actinobacterial members such as those from the genera *Mycobacterium*, *Rhodococcus*, *Gordonia*, *Pseudonocardia*, *Nocardia*, *Marmoricola* and *Actinomycetospora*. Genus *Mycobacterium*-related clones were particularly diverse. Group A-III (genus *Nocardia*) and A-IV (genus *Mycobacterium*) also consisted of *Actinobacteria*-related clones. Group A-V, which was the largest *alkB* clone group (44.5 % of the total) in this study, was affiliated to *Alpha*/*Gammaproteobacteria*-related clones. Additional file 1: Figure S1b shows phylogenetic distribution in group A-V in more detail. The genera *Stenotrophomonas* and *Pseudomonas* of *Gammaproteobacteria*, and genera *Caulobacter* and *Pseudoxanthomonas* of *Alphaproteobacteria*, were the main members of this group. Group A-II included the *alkB* sequences close to those derived from *Betaproteobacteria* (genus *Burkholderia*) and *Gammaproteobacteria* (genera *Nevskia* and *Solimonas*) (Fig. 3). As mentioned above, *Actinobacteria*-related *alkB* clones were distributed in groups A-I, A-III and A-IV. In group A-I, *alkB* genes retrieved from all systems were found (Additional file 1: Figure S1a). However, as well as group A-IV, genus *Mycobacterium*-related *alkB* clones in group A-I were found only in the rhizoplane samples. Group A-III, which included *alkB* genotypes derived from the genus *Nocardia*, was composed of only *alkB* clones (OTU A4) from the rhizoplane of *Z. matrella* (Fig. 3). Group A-V showed a prominent distribution feature (Additional file 1: Figure S1b). Genus *Caulobacter*-related *alkB* clones (OTU A1) were found in considerable numbers in all the rhizoplane samples. Meanwhile, almost all genus *Pseudoxanthomonas*-related *alkB* clones (OTU A2) were found in the unplanted oil-contaminated soil samples. Group A-II included *alkB* clones from all systems (Fig. 3). Genus *Burkholderia*-related *alkB* genotypes (OTU A6) were obtained from all systems. In contrast, genus *Solimonas*-related *alkB* clones (OTU A7) and genus *Nevskia*-related clones (OTU A3) were found in the unplanted oil-contaminated soil samples and the rhizoplane samples, respectively.

**Fig. 4:**
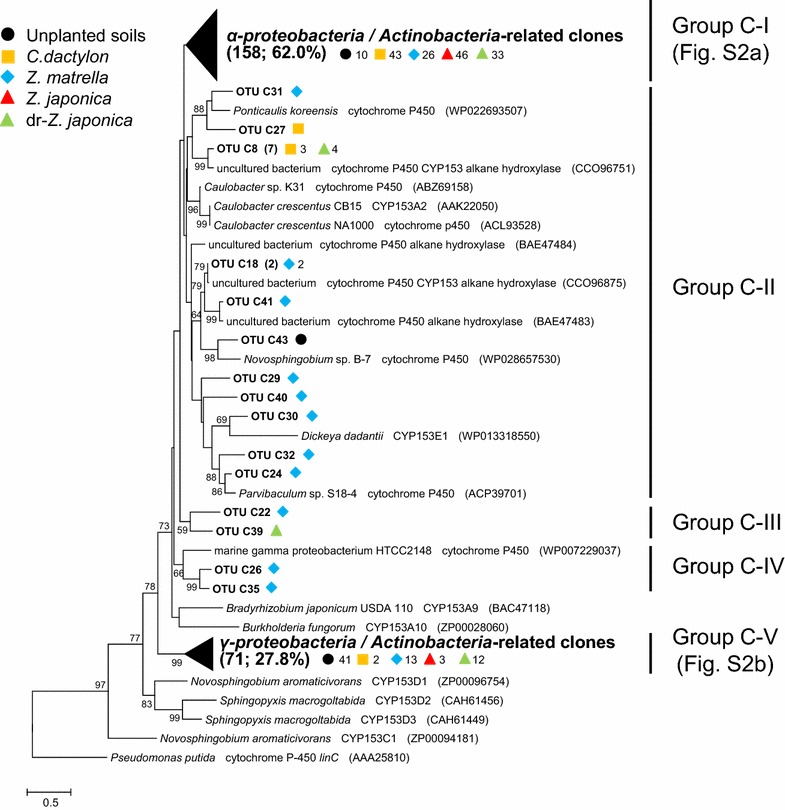
Evolutionary distance dendrogram of retrieved *CYP153* sequences with the reference sequences from NCBI database based on OTU grouping. *Numbers* in *parenthesis* show the numbers of sequences affiliated to the same OTU and group. *Symbols* are used to distinguish different clone libraries. *Numbers* on the *right-hand* of the *symbols* reflect the numbers of sequence within each clone library. Bootstrap values below 50 % are not shown

*CYP153* OTUs were phylogenetically divided into five groups (Fig. 4). Group C-I, which accounted for 62.0 % of total *CYP153* clones, was composed of *Alphaproteobacteria*/*Actinobacteria*-related clones. Additional file 1: Figure S2a shows phylogenetic distribution in group C-I in more detail, indicating that this group was mainly composed of clones affiliated to a wide variety of *Alphaproteobacteria* such as the genera *Bradyrhizobium*, *Afipia*, *Sphingobium*, *Sphingopyxis* and *Parvibaculum*. Group C-V, the second largest *CYP153* clone group (27.8 % of the total) in this study, was affiliated to *Gammaproteobacteria*/*Actinobacteria*-related clones. Additional file 1: Figure S2b shows phylogenetic distribution in group C-V in more detail. In this group, clones closely related to genus *Alcanivorax* in *Gammaproteobacteria* and genus *Aeromicrobium* in *Actinobacteria* were mainly found. Groups C-II and C-IV were composed of *Alphaproteobacteria*‐ and *Gammaproteobacteria*‐related clones, respectively (Fig. 4). Group C-III formed a specific branch distinct from the *CYP153* gene reference sequences. Group C-I was mainly composed of *CYP153* clones derived from the rhizoplane samples (Fig. 4 and Additional file 1: Figure S2a). The genus *Parvibaculum*-related *CYP153* gene clones (OTU C2, OTU C4 and OTU C6), which were the most abundant clustered sequences in group C-I, seemed to be concentrated on the rhizoplane samples other than *Z. matrella*. *CYP153* genes close to the uncultured Rhizobiales bacterium HF4000 48A13 (OTU C3) were also found abundantly on the rhizoplanes. Group C-V was mainly composed of *CYP153* clones derived from the unplanted oil-contaminated soil samples (Fig. 4 and Additional file 1: Figure S2b). In particular, *CYP153* gene clones (OTU C1) close to an uncultured bacterium clone (accession No. BAE47472) were prominently abundant in group C-V. These clones were phylogenetically close to *CYP153* genes derived from the genus *Alcanivorax*, but were clearly of a different genotype. The genus *Aeromicrobium*-related *CYP153* gene clones (OTU C5 and OTU C36) that are affiliated to the phylum *Actinobacteria* were found only on the rhizoplane (Additional file 1: Figure S2b). Finally, groups C-II, C-III and C-IV were mainly composed of *CYP153* gene genotypes from the rhizoplane of *Z. matrella* (Fig. 4).

### Sequence similarities of the *alkB* and *CYP153* genes at amino acid levels to NCBI database

Table 2 shows OTUs with more than 10 % contribution to each of the tested systems. *alkB* and *CYP153* sequences affiliated with OTUs in Table 2 accounted for 68.4 % (186/272) and 65.9 % (168/255), respectively. Genotypes (OTUs) of retrieved alkane hydroxylase genes were apparently different among all systems (Table 2). As *alkB*, the genotypes contained in OTU A2 and A7 were mainly found in the unplanted system, and were similar to *alkB* sequences of *Pseudoxanthomonasspadix* BD-a59 (range of similarity from 96 to 100 %, accession No. WP014160618) and genus *Solimonas* (range of similarity from 95 to 96 %, accession No. WP028007262 and WP020647923), respectively. OTU A1 and A5 were found in all grass-planted systems. OTU A4 (81–83 % similarity to *Nocardia* sp. NRRL WC-3656, accession no. WP030513392), OTU A8 (90–91 % similarity to uncultured bacterium clone, accession no. ACZ64725) and OTU A11 (79–80 % similarity to uncultured bacterium clone, accession no. ABB90683) were specifically detected in *Z. matrella*, *C. dactylon* and dr-*Z. japonica*, respectively.

**Table 2:** Distribution of representative AlkB and CYP153 sequences in each system

	System(s)^a, b^	Closest BLAST match	Range of % ID	Sources^c^	Accession no.
*alkB*					
OTU A1	b, c, d, e	Uncultured bacterium	95–99	Oil reservoir	AGW82865
		Uncultured bacterium	94–99	Soil	AID55555
		Uncultured soil bacterium	99	Pristine and hydrocarbon-contaminated soil	AGQ20909
		*Caulobacter* sp. K31	94	Chlorophenol-contaminated groundwater	YP001672212
OTU A2	a, c	*Pseudoxanthomonas spadix* BD-a59	96–100	Gasoline-contaminated soil	WP014160618
OTU A3	b, d, e	*Nevskia soli*	89–99	Soil	WP029919725
		Uncultured bacterium	89–94	Soil	CCO96572
		Uncultured bacterium	90	Soil	CCO96559
OTU A4	c	*Nocardia* sp. NRRL WC-3656	81–83		WP030513392
OTU A5	b, c, d, e	*Mycobacterium tusciae*	94–96	Granular activated carbon	WP014814636
		*Mycobacterium rufum*	93–94	Soil	KGI67335
		*Mycobacterium chubuense* NBB4	92	Estuarine sediment	ACZ65961
		Uncultured bacterium	92	Soil	AID23719
		Uncultured bacterium	93	Sandy soil	ACZ64758
OTU A7	a	*Solimonas flava*	95–96	Polluted farmland soil	WP028007262
		*Solimonas variicoloris*	95–96	Hexane degrading biofilter	WP020647923
OTU A8	b	Uncultured bacterium	90–91	Sandy soil	ACZ64725
OTU A9	c, d	Uncultured bacterium	79–97	Sandy soil	ACZ64717
OTU A10	a, c, d	Uncultured bacterium	96–97	Soil	AID55553
OTU A11	e	Uncultured bacterium	79–80	Barley field soil	ABB90683
*CYP153*					
OTU C1	a, b, d	Uncultured bacterium	95–96	Crude oil-contaminated soil	BAE47472
OTU C2	a, b, c, d, e	*Parvibaculum lavamentivorans* DS-1	89–100	Activated sludge	WP012110693
		*Parvibaculum lavamentivorans* DS-1	98–99	Activated sludge	YP001413057
OTU C3	a, b, c, d, e	Uncultured Rhizobiales bacterium HF4000_48A13	96–99	Coastal water	ADI19696
		Uncultured bacterium	94–97	Soil	CCO96903
OTU C4	a, b, c, d, e	Uncultured bacterium	83–85	Soil	CCO96723
OTU C5	c, e	*Aeromicrobium marinum*	88–90	Sea water	WP007077898
OTU C6	b, c, d, e	Uncultured bacterium	97–100	Soil	CCO96726
		Alpha proteobacterium MA2	98–99	Marine sediment	GAK46282

^a^System(s) containing the respective OTUs: a, unplanted soil; b, *C. dactylon*; c, *Z. matrella*; d, *Z. japonica*; e, dr-*Z. japonica*

^b^System(s) with high ratios (>10 %) of each OTU were underlined

^c^Source of the corresponding genes from GenPept contains the genes from sole strain and the environmental clones

As *CYP153*, OTU C1, which was the most similar to the uncultured bacterium clone (accession no. BAE47472), indicated a considerable large proportion (76.9 %; 40/52) in the unplanted system. OTU C5 (88–90 % similarity to *Aeromicrobium marinum*, accession no. WP007077898) was found in the *Z. matrella* and dr-*Z. japonica* systems. OTU C4 (83–85 % similarity to the uncultured bacterium clone, accession no. CCO96723) and OTU C6 (97–100 % similarity to *Parvibaculum lavamentivorans* DS-1, accession no. WP012110693) were more abundant in dr-*Z. japonica* and *Z. japonica* systems, respectively. Origins of database sequences with the highest similarity were associated with a range of environments such as oil-contaminated and uncontaminated soils, estuarine and marine sediments and seawater.

### Quantification of two alkane hydroxylase genes

**Fig. 5:**
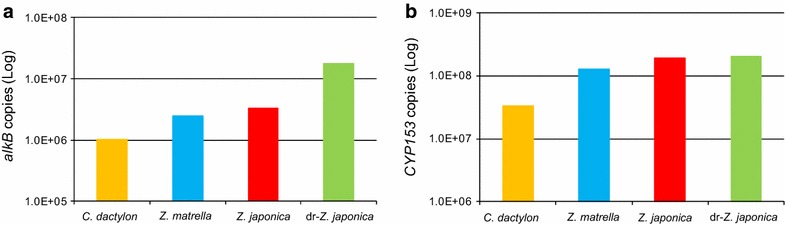
Quantification of alkane hydroxylase genes on the rhizoplanes. **a** *alkB* genes at 856 days (*C. dactylon* and *Z. japonica*) or 891 days (*Z. matrella* and dr-*Z. japonica*) and **b** *CYP153* genes at 494 days

*alkB* and *CYP153* genes in the rhizoplane samples were quantified by qPCR. The copy numbers (copies/g roots) of *alkB* and *CYP153* genes ranged from 1.04 × 10^6^ to 1.79 × 10^7^ copies and 3.29 × 10^7^ to 2.05 × 10^8^ copies, respectively (Fig. 5). The abundances of both alkane hydroxylase genes did not correlate well with degradation efficiencies of TPH (Fig. 1). For instance, an effective decrease in TPH concentration was observed in the *C. dactylon*-planted system, whereas the abundance of both genes on the rhizoplane was lower than in other plants.

## Discussion

Various bacterial phylogenies possess *alkB* and *CYP153* genes, such as *Alpha*-, *Beta*-, *Gamma*- and *Deltaproteobacteria*; *Actinobacteria*; *Bacteroides*; *Firmicutes*; *Spirochetes* and *Planctomycetes* (Wang et al. 2010a, b; Nie et al. 2014a). In the present study, these two genes were also detected in abundance on the rhizoplanes of grasses (Fig. 5). In phylogenetic analyses of these two genes, *Alpha*-, *Beta*-, *Gammaproteobacteria* and *Actinobacteria*-related *alkB* and *CYP153* genes were detected on the rhizoplane of grasses. These results suggest that these phylogenies play an important role in degrading the oil in the contaminated soils on the rhizoplane of grasses during phytoremediation. Culture-dependent methods show that the genera *Bacillus*, *Ochrobactrum*, *Enterobacter*, *Pontola*, *Arthrobacter*, *Rhodococcus*, *Nocardia* and *Pseudoxanthomonas* have been observed on the rhizoplanes of petroleum-contaminated soils, as the alkanes-degrading bacteria (Al-Awadhi et al. 2009). Alkane hydroxylase genes close to those of phylogenies other than those described above (such as the genera *Mycobacterium*, *Nocardia*, *Aeromicrobium*, *Parvibaculum* and *Caulobacter*) were also detected in abundance on the rhizoplanes in this study. Most of the retrieved sequences were also similar to clones derived from other environments such as oil-contaminated soils and estuarine sediments (Table 2). These results show that genotypes of the alkane hydroxylase genes on the rhizoplanes of grasses are more diverse than previously supposed, and the alkane-degrading rhizobacteria do not consist of rhizosphere-specific bacterial assemblages.

Both alkane hydroxylase genes show higher diversity on the rhizoplanes than in unplanted oil-contaminated soils (Fig. 2). *Actinobacteria*-related *alkB* and *CYP153* genes in particular were more diverse on the rhizoplanes than in the unplanted oil-contaminated soils (Figs. 3, 4; Additional file 1: Figures S1 and S2). The *Actinobacteria*-related alkane hydroxylase genes on the rhizoplanes contained the genes phylogenetically close to those of the genera *Pseudonocardia*, *Marmoricola*, *Aeromicrobium*, *Actinomycetospora*, *Mycobacterium*, *Rhodococcus*, *Gordonia* and *Nocardia*. To the best of our knowledge, this is the first study to report that *alkB* and *CYP153*, phylogenetically close to the first four of these genera, have been detected from the rhizoplanes. Whereas it has been previously discussed (Singh et al. 2007) that *Actinobacteria* are not seemed to be dominant in environments with continuous carbon substrate supply such as the rhizospheres of grasses, Smalla et al. (2001) found the abundance of *Actinobacteria* in the rhizosphere. Our results also imply that *Actinobacteria* were among the most diverse phyla on the rhizoplanes of grasses in oil-contaminated soils. Thus, various actinobacterial species might be some of the main contributors in degrading alkanes in contaminated soils during phytoremediation using grasses.

The root-associated bacteria were different from bacterial communities in bulk soils (Grayston et al. 1998), although the reason(s) why diversity of hydrocarbon-degrading genes on the rhizoplane increased are unclear. The genera *Parvibaculum*, *Caulobacter* and *Mycobacterium*, which were likely to be abundant from the detected genotypes of *alkB* and *CYP153* genes on the rhizoplane in this study, can produce biofilms (Schleheck et al. 2000; Smit et al. 2000; Carter et al. 2003; Ojha and Hatfull 2007). Regonne et al. (2013) proposed that formation of bacterial biofilms might be associated with an increase in diversities of the polycyclic aromatic hydrocarbons (PAHs)-specific ring-hydroxylating dioxygenase alpha subunit gene responsible for phenanthrene degradation on a hydrophobic membrane laid in contaminated soils. Biofilms are considered to enhance PAH availability by increasing contact surface areas between bacteria and hydrophobic hydrocarbons (Eriksson et al. 2002). Bacterial communities in the biofilms physically and physiologically benefit each other (Stach and Burns 2002), and our results suggest that formation of biofilms is likely to help to increase genotypic diversity of alkane hydroxylase genes on the rhizoplanes.

The TPH effectively decreased in all grass-planted systems (Fig. 1), in which *alkB* and *CYP153* genes were more diverse than in the unplanted system. However, copy numbers of both genes were not correlated with degradation efficiencies (Fig. 5). Thus, the diversity of alkane hydroxylase genes may enhance phytoremediation efficiency. It was reported that inoculation of elite alkane degraders increased degradation efficiency during phytoremediation (Soleimani et al. 2010; Afzal et al. 2011). The diversification of alkane hydroxylase genes probably increases the probability of elite alkane degraders appearing in the bacterial community on the rhizoplane. Furthermore, the co-existence of *alkB* and *CYP153* genes in a bacterial cell enlarges the range of alkane degradation (Schneiker et al. 2006; Nie et al. 2014b). Nie et al. (2013) reported that homologues of alkane hydroxylase gene in a bacterial cell expressed at different range of alkanes. Our results suggest that the diversity and genotypes of alkane hydroxylase genes on the rhizoplane is significant in influencing alkane degradation efficiency during phytoremediation. However, further studies regarding the gene expression and activity of both alkane hydroxylases and the link between chain length of degradable alkanes and genotypic patterns of both genes is necessary to test this hypothesis.

## Additional file

**Additional file 1:** **Table S1.** PCR conditions used in this study;** Table S2.** Diversity indices of *alkB* and *CYP153* genes in each system;** Figure S1.** Evolutionary distance dendrogram of *alkB* (continuation of Fig. 3). (a) Group AI and (b) Group A-V. Numbers in parenthesis show the numbers of sequences affiliated to the same OTU. Symbols are used to distinguish different clone libraries. Numbers on the right-hand of the symbols reflect the numbers of sequence within each clone library. Bootstrap values below 50% are not shown;** Figure S2.** Evolutionary distance dendrogram of *CYP153* (continuation of Fig. 4). (a) Group C-I and (b) Group C-V. Numbers in parenthesis show the numbers of sequences affiliated to the same OTU. Symbols are used to distinguish different clone libraries. Numbers on the right-hand of the symbols reflect the numbers of sequence within each clone library. Bootstrap values below 50% are not shown.
