# Supplementary material for: Diversity of alkane hydroxylase genes on the rhizoplane of grasses planted in petroleum-contaminated soils
Source: Springerplus. 2015 Sep 18;4:526. doi: 10.1186/s40064-015-1312-0 (PMC4575313; doi:10.1186/s40064-015-1312-0)
Supplement: Supplementary file 1 — Additional file 1: Table S1. PCR conditions used in this study; Table S2. Diversity indices of alkB and CYP153 genes in each system; Figure S1. Evolutionary distance dendrogram of alkB (continuation of Fig. 3). (a) Group AI and (b) Group A-V. Numbers in parenthesis show the numbers of sequences affiliated to the same OTU. Symbols are used to distinguish different clone libraries. Numbers on the right-hand of the symbols reflect the numbers of sequence within each clone library. Bootstrap values below 50% are not shown; Figure S2. Evolutionary distance dendrogram of CYP153 (continuation of Fig. 4). (a) Group C-I and (b) Group C-V. Numbers in parenthesis show the numbers of sequences affiliated to the same OTU. Symbols are used to distinguish different clone libraries. Numbers on the right-hand of the symbols reflect the numbers of sequence within each clone library. Bootstrap values below 50% are not shown. [file 40064_2015_1312_MOESM1_ESM.pdf]

Additional file

Diversity of Alkane Hydroxylase Genes on the Rhizoplane of Grasses Planted in Petroleum-Contaminated Soils

Shun Tsuboi[1,2*]; Shigeki Yamamura[1]; Toshiaki Nakajima-Kambe[3]; Kazuhiro Iwasaki[1]

Affiliation:
[1]National Institute for Environmental Studies (NIES), Center for Regional Environmental Research, 16-2 Onogawa, Tsukuba, 305-8506, Japan
[2]National Institute for Environmental Studies (NIES), Center for Environmental Biology and Ecosystem Studies, 16-2 Onogawa, Tsukuba, 305-8506, Japan
[3]Faculty of Life and Environmental Sciences (Bioindustrial Sciences), University of Tsukuba, 1-1-1 Tennodai, Tsukuba, 305-8572, Japan

*__Corresponding author.__ Shun Tsuboi
E-mail address: tsuboi.shun@nies.go.jp
Telephone numbers: +81-29-850-2204

Table S1 PCR conditions used in this study[a]

Target gene	Objective	Initial denaturation		Denaturing		Annealing		Extension		Final extension		Reference of used primers
		Temp (°C)	Times (s)	Temp (°C)	Times (s)	Temp (°C)	Times (s)	Temp (°C)	Times (s)	Temp (°C)	Times (s)	
alkB	Detection	95	300	94	45	60[b]	45	72	60	72	600	Paisse et al. (2011)
	qPCR	98	120	98	10	55	15	68	30			
CYP153	Detection	95	300	94	45	54	30	72	60	72	600	Wang et al. (2011)
	qPCR	98	120	98	10	54	15	68	60			
almA	Detection	95	300	94	30	50	30	72	60	72	600	Wang and Shao (2012)
ladA	Detection	95	300	94	45	72[c]	60	72	60	72	600	Lo Piccolo et al. (2011)
qPCR standard	Standard preparation	95	600	94	30	55	30	72	60	72	600	

[a] Steps from "Denaturing" to "Extension" were sequentially repearted 35 times in the detection of target genes and the standard create, and 40 times in the qPCR.

[b] The annealing temperature for the PCR decreased from 60 to 50°C at 1°C/cycle and was kept constant at 50°C for the last 25 cycles.

[c] The annealing tempereture for the PCR decreased from 72 to 58 (with a 2°C decremental step from cycle 2 to 8) plus 26 cycles at 56°C constant.

Table S2 Diversity indices of *alkB* and *CYP153* genes in each system

System	*alkB*				*CYP153*			
	# of sequences	# of OTUs	Chao1	Shannon-Weaver (*H'*)	# of sequences	# of OTUs	Chao1	Shannon-Weaver (*H'*)
Unplanted soil	60	10	24.1	1.77	52	9	31.1	1.37
C. dactylon	50	26	53.1	3.22	49	15	23.5	2.72
Z. matrella	56	17	49.7	2.52	52	25	138.4	3.00
Z. japonica	50	11	96.9	1.84	51	12	18.1	2.07
dr-*Z. japonica*	56	17	64.7	2.48	51	16	52.5	2.64

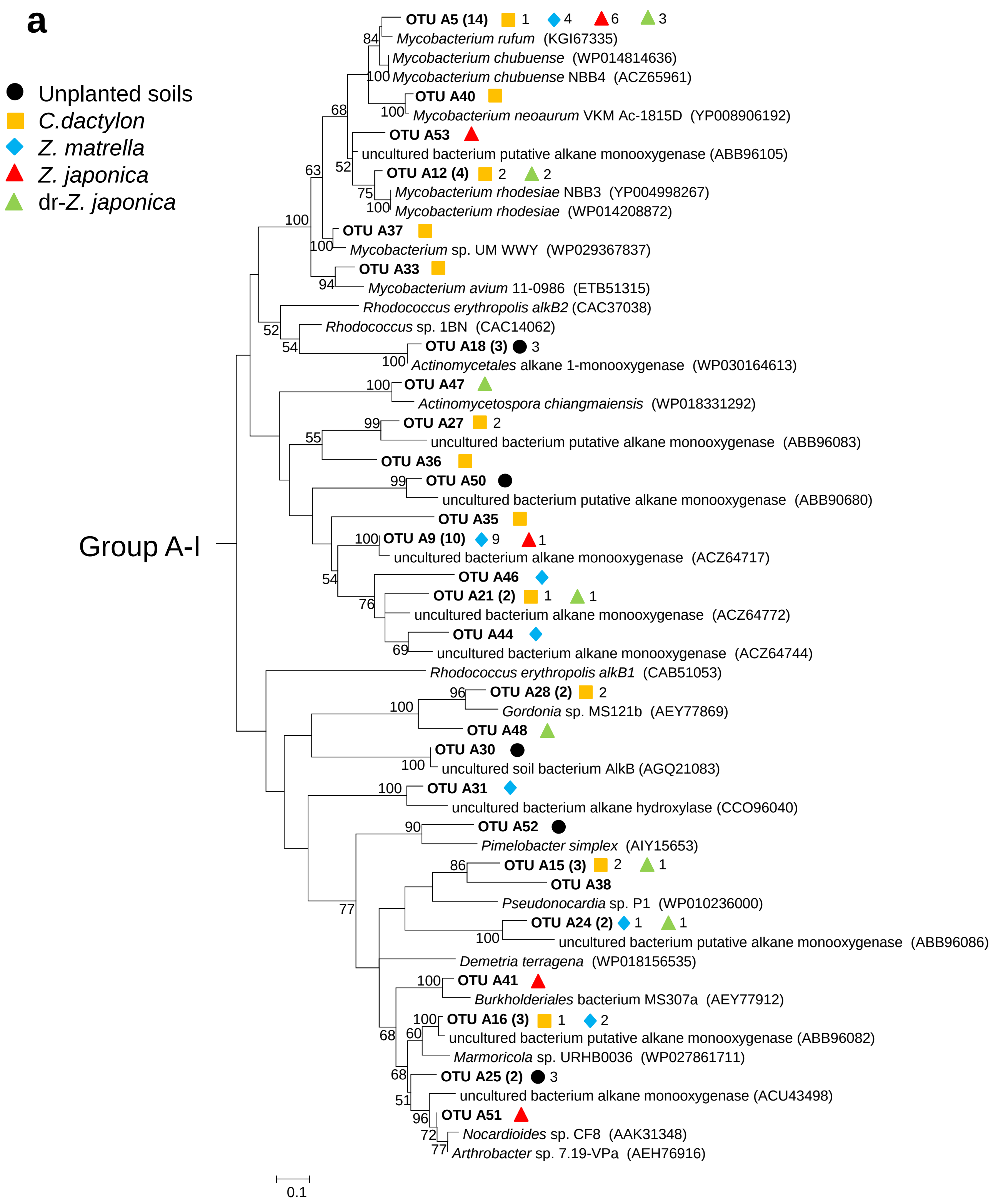

Figure S1 Evolutionary distance dendrogram of *alkB* (continuation of Figure 3). (a) Group A-I and (b) Group A-V. Numbers in parenthesis show the numbers of sequences affiliated to the same OTU. Symbols are used to distinguish different clone libraries. Numbers on the right-hand of the symbols reflect the numbers of sequence within each clone library. Bootstrap values below 50% are not shown

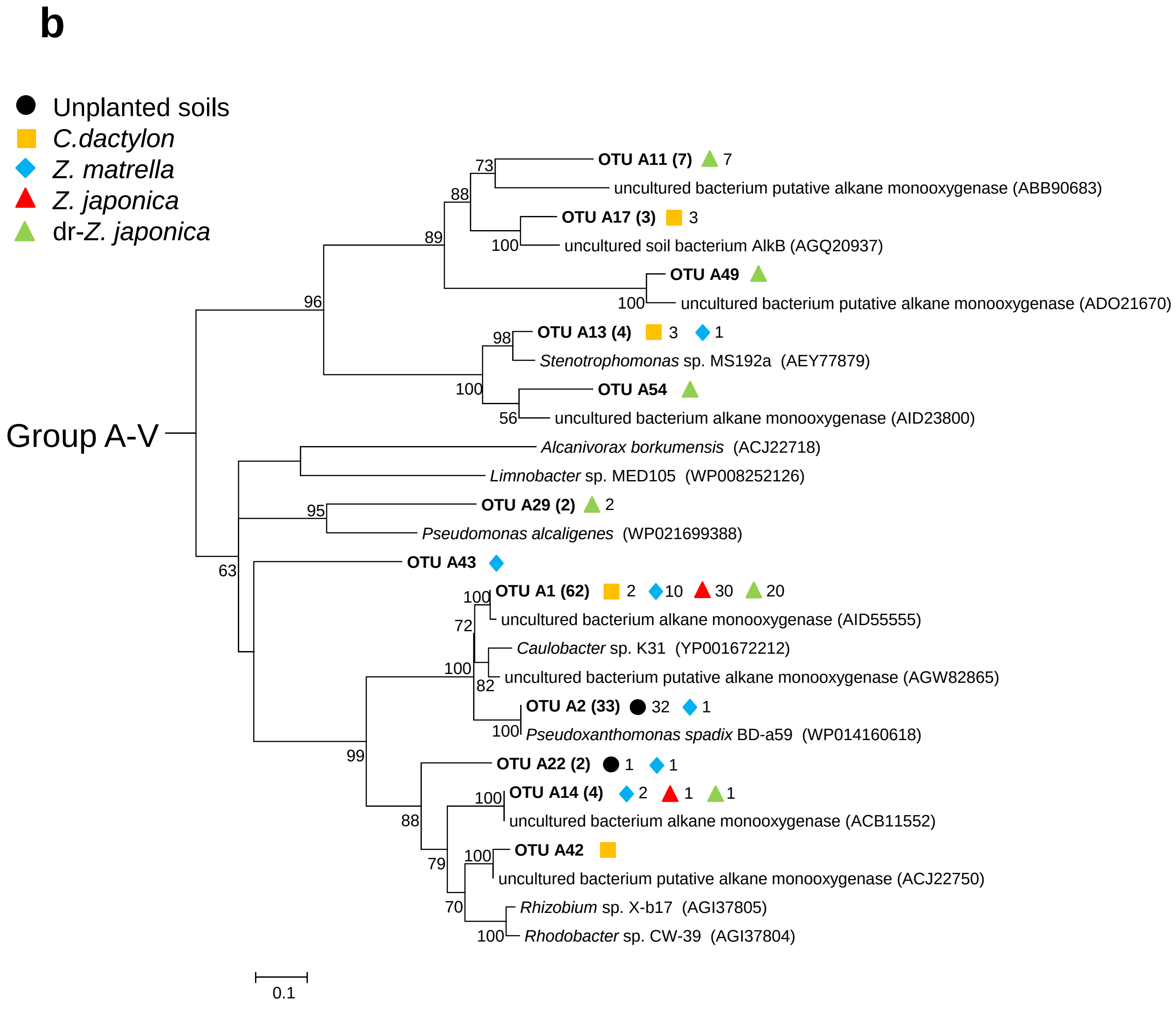

Figure S1 Continuation

a

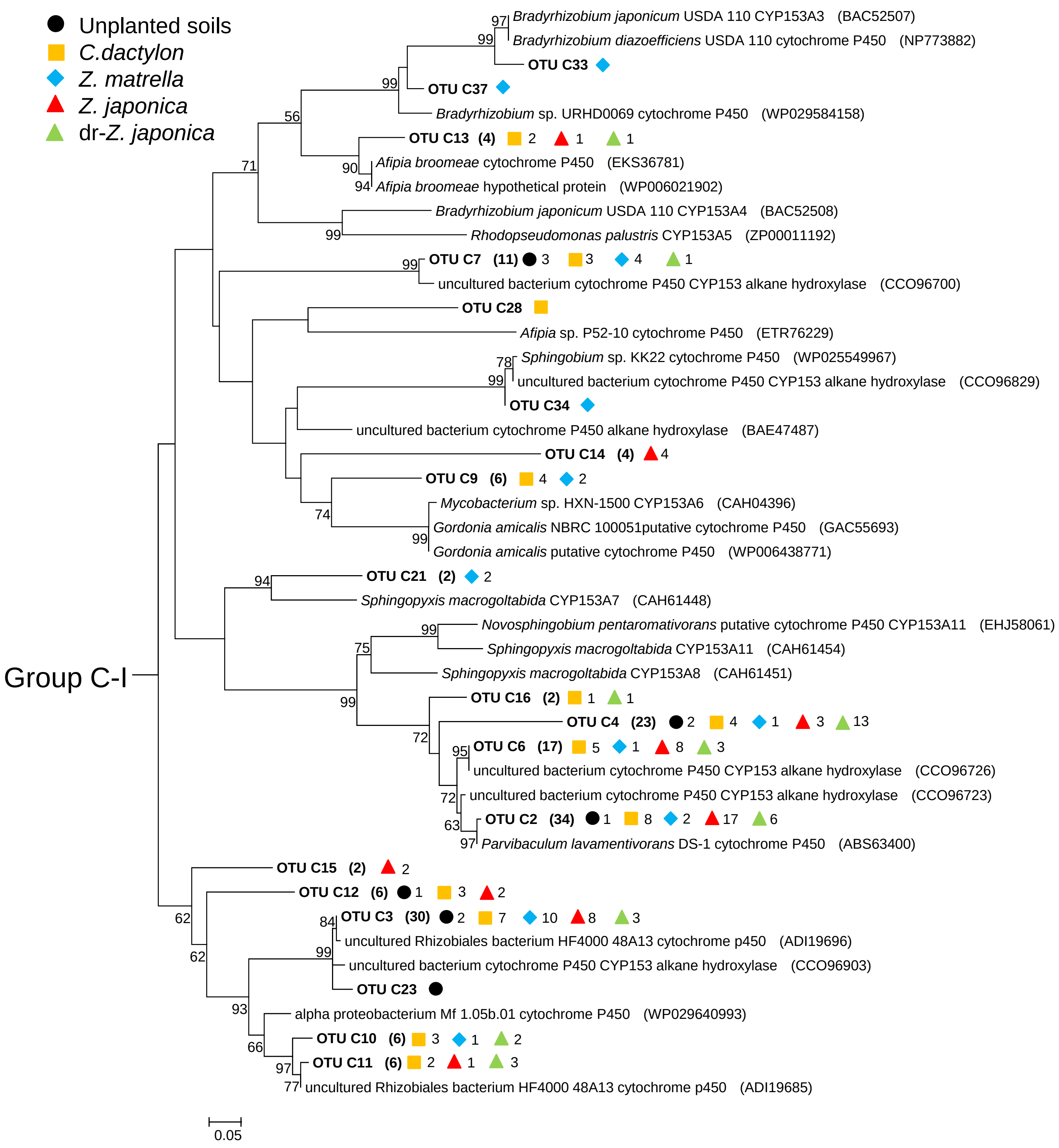

Figure S2 Evolutionary distance dendrogram of *CYP153* (continuation of Figure 4). (a) Group C-I and (b) Group C-V. Numbers in parenthesis show the numbers of sequences affiliated to the same OTU. Symbols are used to distinguish different clone libraries. Numbers on the right-hand of the symbols reflect the numbers of sequence within each clone library. Bootstrap values below 50% are not shown

b

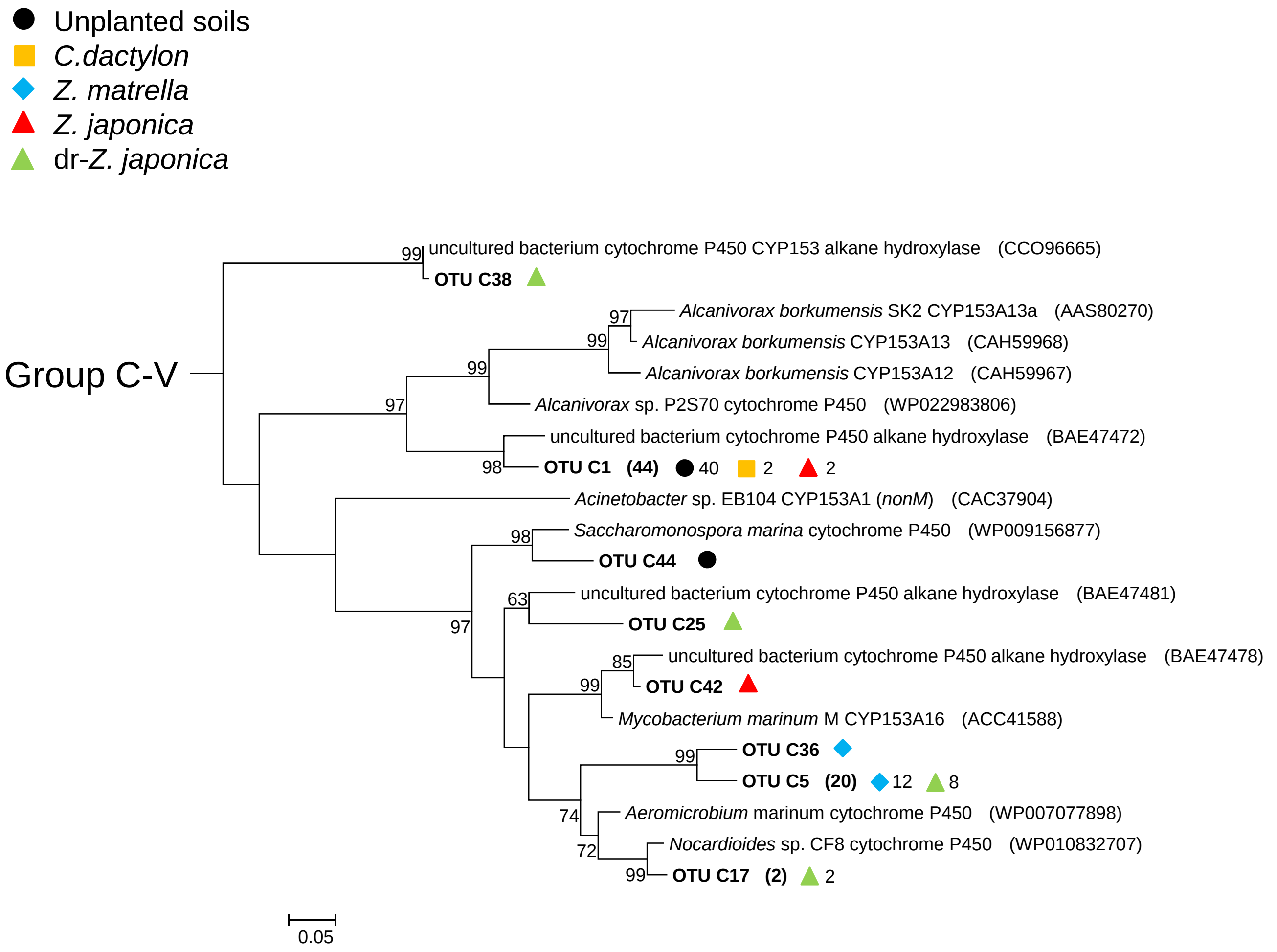

Figure S2 Continuation

References

Lo Piccolo L, De Pasquale C, Fodale R, Puglia AM, Quatrini P (2011) Involvement of an alkane hydroxylase system of *Gordonia* sp. strain SoCg in degradation of solid *n*-alkanes. Appl Environ Microbiol 77:1204–1213

Paisse S, Duran R, Coulon F, Goñi-Urriza M (2011) Are alkane hydroxylase genes (*alkB*) relevant to assess petroleum bioremediation processes in chronically polluted coastal sediments?. Appl Microbiol Biotechnol 92:835–844

Wang W, Shao Z (2012) Diversity of flavin-binding monooxygenase genes (*almA*) in marine bacteria capable of degradation long-chain alkanes. FEMS Microbiol Ecol 80:523-533

Wang XB, Chi CQ, Nie Y, Tang YQ, Tan Y, Wu G, Wu XL (2011) Degradation of petroleum hydrocarbons (C6-C40) and crude oil by a novel *Dietzia* strain. Bioresour Technol 102:7755–7761
